# Recent Developments in Automated Reactors for Plasmonic Nanoparticles

**DOI:** 10.3390/nano15080607

**Published:** 2025-04-15

**Authors:** Shan He, Tong Luo, Xiao’e Chen, David James Young, Matt Jellicoe

**Affiliations:** 1 School of Food and Pharmacy, Zhejiang Ocean University, Zhoushan 316022, China; 2 James Watt School of Engineering, University of Glasgow, Glasgow G12 8QQ, UK; david.j.young@glasgow.ac.uk; 3 College of Medicine and Public Health, Flinders University, Adelaide 5042, Australia

**Keywords:** automated reactors, plasmonic nanoparticles, continuous flow platform

## Abstract

Automated reactors are transforming nanomaterial synthesis by enabling precise, multistep control over morphology and reaction pathways. This review discusses recent advancements in robotic batch and continuous-flow platforms, highlighting their role in expanding chemical space exploration and adaptive manufacturing. Despite progress, challenges remain in integrating automation for complex, multistep syntheses due to the intricate interplay of chemical and physical processes. Emerging process analytical technologies and advanced control software are enhancing real-time monitoring, adaptive feedback loops, and self-optimizing synthesis strategies. We categorize these developments, emphasizing their impact on plasmonic nanomaterial fabrication and outlining future directions for autonomous synthesis.

## 1. Introduction

**Figure 1:**
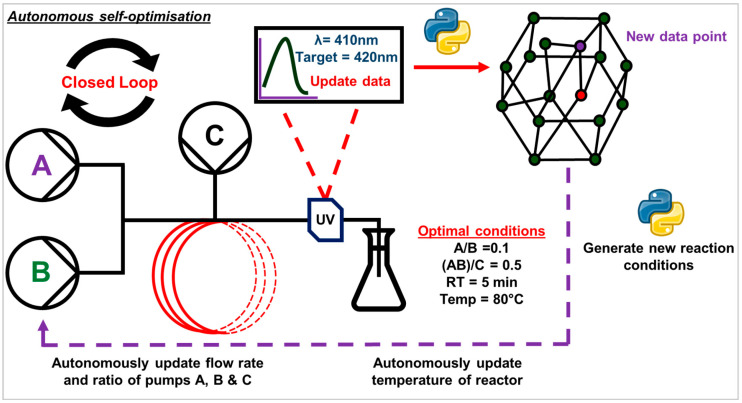
Pictographic representation of a simple autonomous self-optimizing platform using UV-Vis as the inline analysis.

Automated reactors have revolutionized chemical and nanomaterial synthesis by increasing efficiency, precision, and reproducibility [1,2,3,4]. These platforms allow researchers to focus on process optimization and creative problem-solving rather than repetitive manual labor [5]. Plasmonic nanoparticles (PNPs), such as gold and silver, play a crucial role in biomedical engineering, optics, and catalysis, necessitating precise control over their size, shape, and surface properties [6,7,8,9]. While batch synthesis remains widely used, continuous flow platforms offer several advantages, including enhanced reproducibility, scalability, and process optimization [10]. The global nanomaterials market is expected to grow significantly, driven by increasing demand for precision-engineered nanoparticles in various fields such as healthcare, electronics, and sustainable energy solutions [11,12,13]. Autonomous self-optimization of nanoparticle synthesis has emerged as a crucial area of development, allowing researchers to achieve user-defined nanoscale geometries that are otherwise challenging to fabricate [14]. By integrating continuous flow synthesis with advanced automation techniques, researchers can unlock novel synthetic methodologies, ensuring safety, reproducibility, and scalability in nanoparticle manufacturing [10,15] (Figure 1).

Recent advancements in continuous flow reactors have demonstrated their potential in optimizing the synthesis of plasmonic nanoparticles [16]. These platforms enable real-time monitoring, adaptive feedback control, and rapid fine-tuning of reaction parameters to achieve high-quality nanoparticles with tailored optical, electrical, and catalytic properties. Additionally, integrating microfluidic reactors into continuous flow synthesis has significantly improved reaction kinetics, mixing efficiency, and heat transfer, allowing for superior control over nanoparticle morphology and composition [17,18,19,20]. Furthermore, autonomous self-optimization enables safer and more efficient nanoparticle synthesis by incorporating real-time toxicity assessment and Bayesian optimization algorithms [21]. These algorithms iteratively refine reaction conditions based on real-time feedback, enhancing reproducibility while reducing experimentation time [22,23]. This capability is particularly beneficial in biomedical applications, where precise control over nanoparticle properties is essential for drug delivery, biosensing, and imaging applications [24]. There are few examples of the use of automated reactors (batch or continuous flow) being used to synthesize plasmonic nanoparticles. As the field continues to evolve, the integration of AI-driven predictive models and machine learning algorithms will further enhance the efficiency and adaptability of automated reactors [25]. By addressing key challenges such as reactor fouling, process scalability, and inline characterization limitations, future advancements will pave the way for next-generation nanofabrication technologies.

This review explores recent developments in automated reactor technologies over the past 5 years, highlighting their impact on plasmonic nanomaterial synthesis and potential applications in diverse industries.

## 2. Batch Platforms

The advent of batch-based automated reactors has significantly improved the synthesis of PNPs. Batch synthesis remains the most widely used method for nanoparticle production due to its straightforward implementation and scalability. However, the emergence of automation in batch platforms has led to notable advancements in nanoparticle synthesis, enhancing reproducibility and efficiency. The automation of batch reactors primarily focuses on integrating artificial intelligence, real-time feedback mechanisms, and advanced spectroscopic techniques to achieve higher precision.

**Figure 2:**
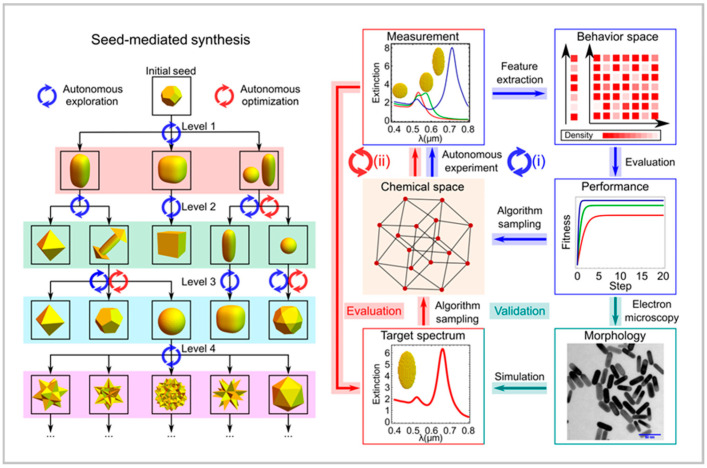
The closed-loop approach toward exploration and optimization in the seed-mediated synthesis of nanoparticles. Reproduced from [14] under a Creative Commons 4.0 CC BY license.

Salley et al. (2020) introduced a genetic algorithm-driven robotic platform for gold nanoparticle synthesis, marking one of the first implementations of self-optimizing batch reactors [26]. This study demonstrated how AI could autonomously adjust reaction conditions based on experimental fitness scores obtained through UV-Vis spectroscopy, ultimately enabling the production of diverse nanoparticle morphologies such as rods and octahedral structures. The success of this approach laid the foundation for the application of evolutionary algorithms in nanomaterial synthesis, showing that AI could not only optimize reaction conditions but also facilitate the discovery of new material structures. Building on this idea, Jiang et al. (2022) expanded the concept of AI-driven nanoparticle synthesis by incorporating theoretical modeling and machine learning to refine nanostructure design further. Their approach integrated real-time spectroscopic feedback and hierarchical machine learning models to optimize nanoparticle formation dynamically, achieving an impressive 95% yield [14] (Figure 2). Unlike Salley et al. [26], who primarily used fitness scores for optimization, Jiang et al. introduced a closed-loop system that continuously refined reaction parameters, significantly improving reproducibility and reducing material waste. This work also introduced a more sophisticated digital documentation approach, using the chemical description language (χDL) to ensure reproducibility and systematic tracking of reaction conditions. The incorporation of real-time optimization and digital synthesis documentation demonstrated a significant leap in the capabilities of autonomous experimental platforms.

**Figure 3:**
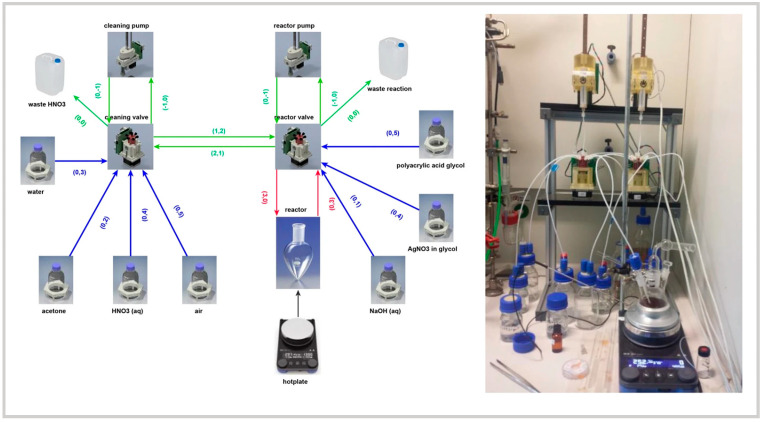
Chemputer schematic (**left**) and physical setup (**right**) showing components, metadata, and tubing connections for automated nanoparticle synthesis. Reproduced from [27] under a Creative Commons 4.0 CC BY license.

Parallel to these advances, Wolf et al. (2022) focused on the automation of a specific synthesis process—the polyol method for silver nanoparticles [27] (Figure 3). Rather than emphasizing AI-driven optimization, their study showcased how automation alone could lead to improved reproducibility and scalability in colloidal synthesis. By integrating small-angle X-ray scattering and dynamic light scattering techniques, their platform successfully produced silver nanoparticles with highly controlled sizes (3 and 5 nm). This work highlighted a key challenge in automated synthesis: predefined reaction conditions can sometimes limit adaptability to variations in synthesis environments. Compared to Jiang et al. and Salley et al., who focused on AI-enhanced self-optimization, Wolf et al.’s work emphasized the role of automation in maintaining consistency in well-established chemical processes.

More recently, Yoo et al. (2024) advanced the AI-driven approach further by implementing a Bayesian optimization model coupled with a UV-Vis spectroscopy module, enabling rapid optimization of silver nanoparticle synthesis at room temperature within just 200 iterations [28]. This work refined the AI-driven synthesis process introduced by Salley et al. and Jiang et al. by incorporating a self-adjusting mechanism capable of adapting in real time to changing synthesis parameters. While their study provided an efficient optimization framework, its reliance on a single analytical technique limited its real-time monitoring capabilities, echoing some of the challenges faced by Wolf et al. Despite this limitation, Yoo et al.’s work further demonstrated the power of AI-driven autonomous synthesis by reducing the complexity and cost of nanoparticle production, making it more accessible and efficient.

Despite these advancements, automated batch synthesis platforms face several challenges. One significant drawback is the need for intermittent human intervention to handle unexpected variables such as impurity accumulation, inconsistent precursor quality, or undesired side reactions. Additionally, while parallel combinatorial experimental techniques have been applied to nanomaterials, they have not yet been effectively integrated into autonomous experimentation systems. Another limitation is the lag time in sample characterization, which, despite real-time analytical tools, still requires validation through offline techniques such as electron microscopy. The accumulation of byproducts and the need for extensive purification steps also contribute to increased waste and environmental impact, making continuous flow platforms an attractive alternative (Table 1).

## 3. Continuous Flow Platforms

Continuous flow synthesis has revolutionized nanofabrication by providing enhanced control over reaction conditions, enabling inline analysis, and ensuring precise reagent handling [35,36]. Unlike batch processing, which often faces challenges with inconsistencies in particle size, morphology, and reproducibility, continuous flow methods utilize controlled flow rates, residence times, and reaction environments to achieve superior uniformity and scalability [37,38,39]. Despite these advantages, challenges such as energy consumption, reactor clogging, and the integration of real-time analytical techniques persist. This section critically evaluates key studies that have advanced the field of continuous flow nanomaterial synthesis.

**Figure 4:**
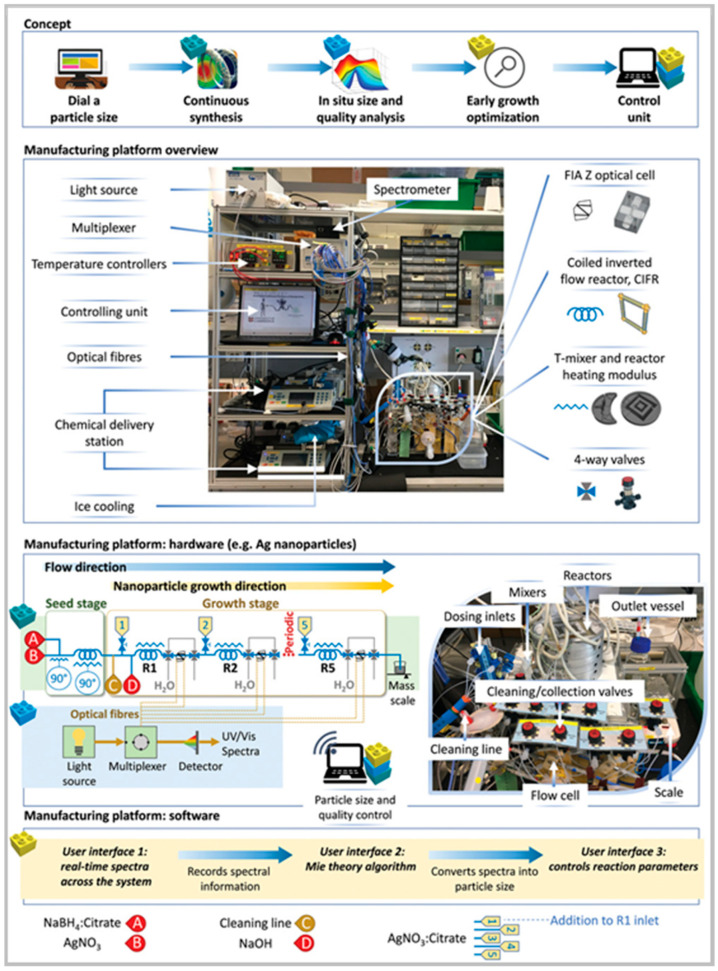
Autonomous self-optimization of specific size Au and AgNPs. Reproduced from [29] under a Creative Commons 4.0 CC BY license.

Pinho and Torrente-Murciano (2021) introduced the “Dial-a-Particle” system, a microfluidic reactor platform designed for the precise manufacturing of plasmonic nanoparticles (Figure 4) [29]. This innovative system combines fast, integrated multipoint particle sizing with a modular “plug-n-play” platform, featuring reactors in series and distributed feed capabilities. The real-time early growth information obtained allows for accurate prediction and control of particle properties, enabling automated synthesis of nanoparticles with tunable sizes ranging from approximately 4 to 100 nm. This approach represents a significant advancement toward reproducible nanomaterial production. However, the study primarily utilized UV-Vis spectroscopy for characterization, which offers limited insight into detailed morphological features. Future work could benefit from integrating advanced analytical techniques, such as electron microscopy or dynamic light scattering, to enhance characterization accuracy.

**Figure 5:**
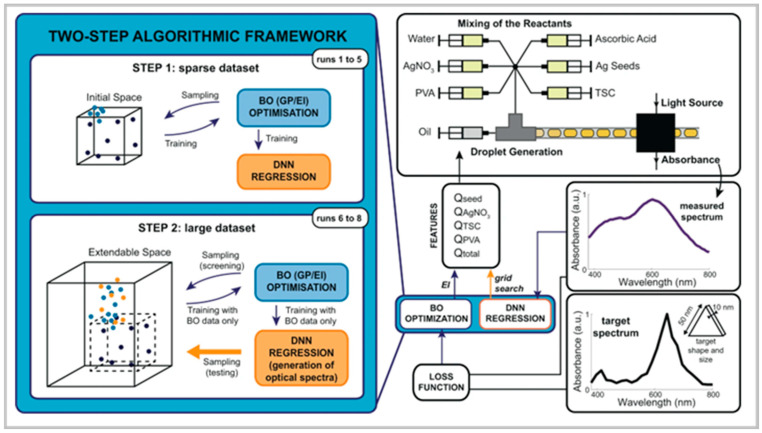
Autonomous self-optimization of AgNPs through a two-step optimization algorithmic framework. Reproduced from [30] under a Creative Commons 4.0 CC BY license.

Mekki-Berrada et al. (2021) proposed a two-step machine learning framework for the high-throughput microfluidic synthesis of silver nanoparticles with desired optical properties (Figure 5) [30]. The approach combines Gaussian process-based Bayesian optimization with a deep neural network, enabling the rapid production of silver nanoparticles tailored to specific absorbance spectra. While this method effectively optimized particle shape and size, it required extensive data acquisition prior to model training, presenting a considerable drawback. This study highlights the classic trade-off in machine learning-based synthesis: large datasets improve predictive accuracy but can slow down the optimization process. Future advancements could focus on transfer learning or active learning strategies to reduce the data acquisition burden while maintaining model performance.

Hall et al. (2021) demonstrated the integration of autonomous optimization within a continuous flow system for nanoparticle-catalyzed reactions [31]. They developed an automated continuous flow reactor equipped with inline analysis, applying it to the self-optimization of a gold nanoparticle-catalyzed 4-nitrophenol reduction reaction. The system optimized experimental conditions to achieve maximum conversion in under 2.5 h. Data obtained from this optimization facilitated the generation of a kinetic model, allowing for the prediction of reaction outcomes under varying conditions. This study exemplifies the potential of AI-driven synthesis for catalytic applications, particularly in dynamically optimizing reaction conditions. However, it also underscores a critical bottleneck: the necessity for advanced inline analytical techniques to complement AI-driven decision-making. Without robust real-time monitoring, the system’s ability to make precise adjustments is constrained, limiting its broader applicability.

**Figure 6:**
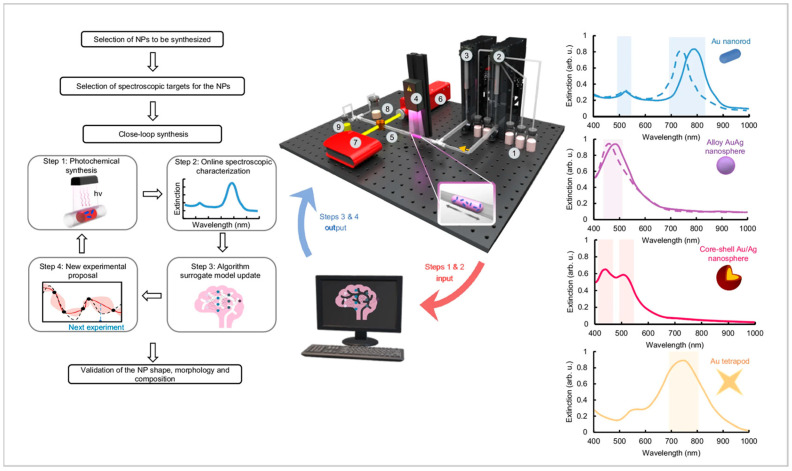
The autonomous fluidic identification and optimization nanochemistry (AFION) self-driving lab showing the schematic of the AFION lab. The lab includes (1) reservoirs with reagent solutions, (2) a pump dispenser to generate a slug of reagent solution, (3) an oscillator pump, (4) a UV light source with adjustable height, (5) flow cell for optical NP characterization, (6) tungsten halogen UV light source, (7) an in-line, fiber-optics, charge-coupled spectrometer, (8) a reservoir for the collection of waste, and (9) a reservoir for NP collection for further characterization. The extinction spectra of synthesized NPs from the AFION self-driving lab. Reproduced from [32] under a Creative Commons 4.0 CC BY license.

Wu et al. (2025) introduced a self-driving laboratory designed for the photochemical synthesis of plasmonic nanoparticles with specific structural and optical characteristics (Figure 6) [32]. This autonomous system integrates real-time monitoring and adaptive feedback mechanisms to fine-tune reaction parameters, ensuring the production of nanoparticles that meet predefined criteria. The study highlights the potential of combining artificial intelligence with photochemical processes to achieve precise control over nanoparticle synthesis, paving the way for advancements in materials science and nanotechnology. However, the implementation of such autonomous systems necessitates sophisticated inline analytical tools capable of providing accurate, real-time data to inform the AI-driven adjustments. The development and integration of these advanced analytical techniques remain a significant challenge, critical for the broader application of self-optimizing synthetic platforms.

Tao et al. (2021) developed a self-driving platform that integrates oscillatory microfluidics, online spectroscopy, and machine learning for the autonomous synthesis of metal nanoparticles [33]. This innovative system employs machine learning algorithms to analyze real-time spectroscopic data, enabling the dynamic adjustment of synthesis parameters to achieve desired nanoparticle properties without human intervention. The study demonstrates the platform’s capability to efficiently navigate complex reaction spaces, optimizing conditions to produce nanoparticles with specific characteristics. This approach not only accelerates the discovery and development of new nanomaterials but also enhances reproducibility in nanoparticle synthesis. However, the successful implementation of such autonomous systems relies heavily on the integration of advanced inline analytical techniques that provide accurate, real-time data. Ensuring the precision and reliability of these analytical components is crucial for the system’s ability to make informed decisions during the synthesis process.

Bui et al. (2024) introduced an automated flow chemistry system employing proportional–integral (PI) feedback control to synthesize silver–gold (AgAu) alloy nanoboxes with precise optical properties [34]. This system utilizes a PI control algorithm based on a first-order plus dead-time model, correlating precursor flow rates with the maximum absorbance peaks of the resulting nanoboxes. By iteratively adjusting the flow rate in response to real-time UV–vis absorbance measurements, the system achieves the target optical characteristics of the AgAu nanoboxes. This approach enhances the consistency and reliability of nanoparticle synthesis, minimizing human intervention. However, the effectiveness of this automated system depends on the accuracy of real-time analytical measurements and the robustness of the feedback control algorithm, which are critical for maintaining the desired product specification.

Collectively, these studies underscore the transformative potential of continuous flow synthesis in nanomaterial fabrication. The integration of real-time monitoring, machine learning, and autonomous optimization not only enhances precision and reproducibility but also addresses scalability and efficiency challenges. Ongoing research focusing on overcoming existing limitations, such as reactor design optimization and advanced inline analytical integration, will be pivotal in fully realizing the capabilities of continuous flow nanomanufacturing.

## 4. Challenges and Future Outlook

The future of automated plasmonic nanoparticle synthesis lies in the integration of advanced machine learning algorithms and real-time adaptive control mechanisms. AI-driven predictive models will enable researchers to fine-tune reaction conditions dynamically, leading to improved process efficiency and reduced material wastage. Additionally, hybrid systems that combine the strengths of batch and continuous flow platforms could provide greater flexibility for complex nanoparticle synthesis. Another promising area is the expansion of inline characterization techniques. While UV-Vis spectroscopy is widely used, the incorporation of complementary methods such as Raman spectroscopy, mass spectrometry, and electron microscopy (in situ liquid TEM) will allow for a more comprehensive understanding of nanoparticle properties [40]. This will enhance the ability to produce nanoparticles with tailored optical, electronic, and catalytic properties. Scalability remains a significant challenge for both batch and continuous flow synthesis. While continuous flow systems offer inherent scalability advantages, further advancements in modular reactor design and process standardization will be required for industrial-scale implementation. Collaborative efforts between academia and industry will be crucial in bridging this gap, ensuring that automated nanoparticle synthesis technologies are both practical and commercially viable.

## 5. Conclusions

In conclusion, automated reactors are transforming the field of plasmonic nanoparticle synthesis, with batch and continuous flow systems offering unique advantages and challenges. While batch platforms have demonstrated high-throughput capabilities, continuous flow methods provide superior reproducibility and real-time optimization. Integrating AI, machine learning, and advanced analytical tools will further enhance the potential of these systems, paving the way for next-generation nanofabrication technologies.

## Figures and Tables

**Table 1:** Summary of automated batch and continuous flow platforms for the synthesis of plasmonic nanoparticles.

Reactor Type	Nanoparticles (NPs)	Morphology (Size and Shape)	Analysis (Online/Offline)	Advantages	Disadvantages	Reference
**Batch Platforms**						
Chemputer-Based Batch Reactor	Silver	3–5 nm, Spherical	Offline (Small-angle X-ray scattering, Dynamic Light Scattering)	High precision, integration of Chemputer for automation	Relies on predefined reaction conditions, limited adaptability	Wolf et al., 2022 [27]
AI-Guided Batch Reactor	Silver	18–32 nm, Spherical and Prisms	Online (UV-Vis Spectroscopy)	AI-guided self-optimization, rapid adaptation to conditions	Limited by reliance on a single analytical technique	Yoo et al., 2024 [28]
Genetic Algorithm Batch Reactor	Gold	10–80 nm, Rods, octahedral structures	Online (UV-Vis Spectroscopy)	Genetic algorithm-driven self-optimization, diverse morphologies	Requires extensive experimental iterations for optimization	Salley et al., 2020 [26]
Machine Learning-Assisted Batch Reactor	Gold	2–100 nm, Multiple shapes	Online (Real-time spectroscopic feedback)	High reproducibility, AI-assisted optimization	Need for additional offline validation (e.g., electron microscopy)	Jiang et al., 2022 [14]
**Continuous Flow Platforms**						
Microfluidic Continuous Flow Reactor	Gold, Silver	4–100 nm, Spherical	Inline (UV-Vis Spectroscopy)	Modular plug-and-play system, rapid parameter adjustments	Limited morphological insights, lacks high-resolution characterization	Pinho and Torrente-Murciano, 2021 [29]
Bayesian Optimization Continuous Flow Reactor	Silver	20–70 nm, Prisms	Inline (UV-Vis Spectroscopy)	Fast, high-throughput, data-driven synthesis	Requires extensive data collection for training	Mekki-Berrada et al., 2021 [30]
Catalytic Continuous Flow Reactor	Gold	11–22 nm, Spherical	Inline (UV-Vis Spectroscopy)	AI-driven optimization of catalytic efficiency, real-time feedback	Limited inline techniques for broader characterization	Hall et al., 2021 [31]
Self-Driving Photochemical Flow Reactor	Gold, Silver	6–92 nm, Multiple shapes	Inline (UV-Vis Spectroscopy excitation)	Photochemical synthesis integration, AI-driven optimization	Requires sophisticated inline analytical tools	Wu et al., 2025 [32]
Oscillatory Microfluidics Flow Reactor	Metal Nanoparticles	12–60 nm, Spherical	Online (Machine learning + Spectroscopy)	Fully autonomous parameter adjustment, rapid material discovery	High computational demand, complex instrumentation	Tao et al., 2021 [33]
Proportional–Integral Feedback Flow Reactor	Ag/Au Alloy	40–60 nm, Cubes	Online (Proportional–Integral feedback, UV-Vis Spectroscopy)	High reproducibility, precise optical tuning	Performance depends on real-time analytical accuracy	Bui et al., 2024 [34]
